# A novel method for isolation and culture of primary swine gastric epithelial cells

**DOI:** 10.1186/s12860-020-00341-7

**Published:** 2021-01-06

**Authors:** Henry Bautista-Amorocho, Jorge Alexander Silva-Sayago, Diego A. Goyeneche-Patino, Tania Liseth Pérez-Cala, Fabio Macías-Gómez, Juan Carlos Arango-Viana, Alonso Martínez

**Affiliations:** 1 Grupo de Investigación en Manejo Clínico CliniUDES, Facultad de Ciencias de la Salud, Universidad de Santander - UDES, Bucaramanga, Colombia; 2 Grupo Bacterias & Cáncer, Departamento de Microbiología y Parasitología, Facultad de Medicina, Universidad de Antioquia, Medellín, Colombia; 3 Grupo de Investigación GICA-UDES, Programa de Medicina Veterinaria, Universidad de Santander, Bucaramanga, Colombia; 4 Maestría en Odontología, Facultad de Odontología, Universidad de Santo Tomás, Floridablanca, Colombia; 5 Grupo de Investigación Biología y Clínica, Departamento de Patología, Facultad de Medicina, Universidad de Antioquia, Medellín, Colombia

**Keywords:** Cell culture, Swine gastric epithelium, Tissue engineering, Animal models, Biotechnology

## Abstract

**Background:**

Culturing primary epithelial cells has a major advantage over tumor-derived or immortalized cell lines as long as their functional phenotype and genetic makeup are mainly maintained. The swine model has shown to be helpful and reliable when used as a surrogate model for human diseases. Several porcine cell lines have been established based on a variety of tissues, which have shown to extensively contribute to the current understanding of several pathologies, especially cancer. However, protocols for the isolation and culture of swine gastric epithelial cells that preserve cell phenotype are rather limited. We aimed to develop a new method for establishing a primary epithelial cell culture from the fundic gland region of the pig stomach.

**Results:**

Mechanical and enzymatic dissociation of gastric tissue was possible by combining collagenase type I and dispase II, protease inhibitors and antioxidants, which allowed the isolation of epithelial cells from the porcine fundic glands showing cell viability > 90% during the incubation period. Gastric epithelial cells cultured in RPMI 1640, DMEM-HG and DMEM/F12 media did not contribute enough to cell adhesion, cluster formation and cell proliferation. By contrast, William’s E medium supplemented with growth factors supports confluency and proliferation of a pure epithelial cell monolayer after 10 days of incubation at 37 °C, 5% CO2. Mucin-producing cell phenotype of primary isolates was confirmed by PAS staining, MUC1 by immunohistochemistry, as well as the expression of *MUC1* and *MUC20* genes by RT-PCR and cDNA sequencing. Swine gastric epithelial cells also showed origin-specific markers such as cytokeratin cocktail (AE1/AE3) and cytokeratin 18 (CK-18) using immunohistochemical and immunofluorescence methods, respectively.

**Conclusions:**

A new method was successfully established for the isolation of primary gastric epithelial cells from the fundic gland zone through a swine model based on a combination of tissue-specific proteases, protease inhibitors and antioxidants after mechanical cell dissociation. The formulation of William’s E medium with growth factors for epithelial cells contributes to cell adhesion and preserves functional primary cells phenotype, which is confirmed by mucin production and expression of typical epithelial markers over time.

## Background

Swine are considered to be one of the most valuable animal models used in preclinical studies due to their physiological and anatomical similarities to those of humans [1, 2]. The swine model has been extensively used for understanding the pathophysiology of diabetes and coronary artery disease (CAD) associated with atherosclerosis and hypercholesterolemia, considering their similarities in terms of cardiovascular anatomy and lipid metabolism profiles between both species [3]. This model has also high-throughput DNA sequencing homology and a chromosome structure similar to that of humans. Therefore, new proteomic and genomic approaches to evaluate malignant tumors using the swine model are rapidly increasing [2, 4–6]. In addition, cancer biology research builds on relevant biological models to explain the multistep process of tumorigenesis in humans. Unlike murine models, the effect of human oncogenic mutations on cell growth, differentiation and apoptosis are similar in primary porcine cells. The swine model has been widely used in the development of lymphoma and osteogenic tumors for the induction of tumorigenesis in primary hematopoietic and fibroblast cells, accordingly [4, 7, 8].

Gastrointestinal disorders are a common reason for economic losses in the swine industry worldwide [9, 10]. For example, non-glandular gastric ulcers are a common pathology with a global prevalence of 93% [11] in this species. Disease outcomes are related to several clinical symptoms due to gastrointestinal bleeding such as anemia and weight loss, perforated ulcers (including peritonitis), and sudden death [10, 11]. However, the pathogenesis of porcine gastric ulceration remains poorly understood. Dietary particle size, feeding strategies, genetic background, hormonal changes, gastric microbiota composition and infectious agents such as *Helicobacter suis* have been suggested to be involved [12]. *H. suis* mainly colonizes the gastric fundic gland region causing chronic inflammation [11]. Several studies have found a strong association between the presence of *H. suis* in this glandular area and the prevalence and severity of lesions in the non-glandular area [11, 13, 14]. Therefore, the use of primary swine gastric epithelial cells from the fundic gland region may shed light on the biology involved in the development of this multifactorial disease.

Primary swine cell lines have also been established from different body tissues, including mammary glands [5], kidneys [6], small intestine [15], trachea [6], lungs [16], and alveoli [17]. These primary cells have been used to evaluate gene expression patterns, drug susceptibility and cell physiology [18]. However, as for the pig stomach, the protocols for isolation and culture of gastric cells that combine different approaches and preserve epithelial cell phenotype [19–22] have been described in a few cases. Therefore, this research is aimed to develop a new method for establishing a primary cell culture derived from the fundic gland region of the porcine stomach. The protocol uses mechanical and enzymatic dissociation and optimizes culture conditions to maintain high cell viability and epithelial cell phenotype. This new cell culture method for the isolation of normal gastric epithelial cells is suggested as a model for studying gastric pathologies in humans and swine.

## Methods

### Animal sample collection

Animal experiments were approved by the Ethics Committee on Animal Experimentation of the University of Antioquia under approval number 121/2018, according to the Colombian Regulations for the Use of Laboratory Animals in Biomedical Research (Law 8430 of 1993 and Law 84 of 1989). Fresh stomach tissues were obtained from three young-adult male pigs (*Sus scrofa domesticus*) at ages of 30–34 weeks and an average weight of 80 kg (± 8 kg). These tissues were kindly donated by the owner of a private pig farm that has a local slaughterhouse named “Vijagual”. The approval for the use of pig tissue in this research was obtained through written informed consent by the farm owner. Animals were in good body conditions and disease-free, according to the veterinarian responsible for food safety and hygiene of the above slaughterhouse. About 25–100 g from the fundic gland region of the pig stomach were dissected and stored at 4 °C in 50 mL conical centrifuge tubes for viral transport medium, which contained DMEM-HG (catalog number: 12100061, GIBCO, USA) supplemented with 200 U/mL penicillin, 200 μg/mL streptomycin (catalog number: 15140122- GIBCO, USA), 100 μg/mL gentamicin (catalog number: 15750078 - GIBCO, USA) and 5 μg/mL amphotericin B (catalog number: 15290018 - GIBCO, USA). Tissue samples were immediately shipped to the Biomedical Research Laboratory of the University of Santander - UDES.

### Composition of the digestion medium

The digestion medium for tissue disaggregation and cell detachment contained a Hank’s Balanced Salt Solution (HBSS) with calcium and magnesium (HBL03-Caisson, Smithfield, USA), supplemented with 200 U/mL collagenase type I (C0130-Sigma-Aldrich, St. Louis, USA), 1.2 U/mL dispase II (D4693-Sigma-Aldrich, St. Louis, USA), 0.01 mg/mL soybean trypsin inhibitor (STI) (29129-Chem Cruz, Dallas, USA), 1.25 mg/mL bovine serum albumin (BSA) (B005-Caisson, Smithfield, USA) and 0.1 mM Dithiothreitol (DTT) (A2948- PanReac, Barcelona, Spain). The solution was freshly prepared, filtered with 0.2 μM nylon membranes, stored at 4 °C and used within the next 24 h.

### Composition of the proliferation medium

Different culture media preparations were evaluated to establish the best conditions for the isolation and growth of Gastric Epithelial Cells (GEC) in vitro. DMEM-HG, DMEM/F12 (DFP02-Caisson, Smithfield, USA), RPMI 1640 (catalog number: 11875119, GIBCO, USA) and William’s E (WML01-Caisson, Smithfield, USA) media were supplemented with 20% heat-inactivated fetal bovine serum (FBSi) (026–100- Cell Application, San Diego, CA, USA), 2.5 μg/mL amphotericin B, 100 U/mL penicillin, 100 μg/mL streptomycin, 25 μg/mL gentamicin, 1% L- glutamine (GLL01-Caisson, Smithfield, USA), 25 mM HEPES (4-(2-hydroxyethyl)-1-piperazineethanesulfonic acid) (IVL01-Caisson, Smithfield, USA), 50 ng/mL epidermal growth factor (EGF) (RP1026AF- Cell Application, San Diego, CA, USA) and 4 μg/mL insulin (Apidra D-65926 -Sanofi-Aventis, Germany). The solutions were freshly prepared and filtered with 0.2 μM nylon membranes and stored at 4 °C.

### GEC isolation and culture

Fundic glandular tissues from the pig stomach (*n*=3) were transferred into 100-mm cell culture plates containing 10 mL of fresh transport medium. Fat, connective and muscular tissues were mechanically removed by dissecting the mucous membrane layer (epithelium) using sterile tweezers and surgical scissors. The epithelial layer of the mucous membrane was peeled off through gentle scraping. Tissues were sectioned into pieces of approximately 1 mm^3^ in size, later transferred to 50 mL conical tubes and centrifuged at 80 g and 4 °C for 10 min. Supernatants were discarded and tissue fragments were resuspended in the digestion medium, being constantly agitated in an orbital shaker at 150 rpm and 37 °C for 2 h. After that, the resulting cell suspension was filtered through sterile gauze, rinsed three times to remove residual mucus and centrifuged at 80 g for 10 min. Supernatants were carefully discarded again so that GEC viability could be determined using the trypan blue dye exclusion test (T8154-Sigma-Aldrich, St. Louis, USA). Cells were seeded at a density of 3.5 × 10^5^ cells/well in the proliferation medium into plastic 12-well plates that had been previously treated with a 400 μL bovine collagen type I coating solution (125–50 -Sigma-Aldrich, St. Louis, USA). Cells were incubated at 37 °C in a humidified atmosphere containing 5% CO_2_ for 24 h. Non-adherent cells were removed by washing each well twice with pre-warmed HBSS the next day. Then, a fresh proliferation medium was immediately added and replaced every other day. By the eighth day of incubation, the proliferation medium was reduced to half of the initial concentration of EGF (25 ng/mL), insulin (2 μg/mL) and FBSi (10%). To avoid fibroblast contamination, the initial GEC monolayer was passaged with differential trypsinization strategies based on Jones’ protocol with some modifications [23]. Cell cultures were washed once in PBS 1X and incubated with a 0.25% Trypsin/EDTA solution at 37 °C for 3 min. Weakly adherent cells (fibroblasts) were immediately removed by discarding supernatants. GECs remained attached to the culture surface being later detached using a second Trypsin/EDTA solution for 5 min under the same conditions. Culture plates were thoroughly observed under a light microscope to ensure that only cells showing an epithelial phenotype remained attached after the initial trypsinization before subculturing.

### GEC growth rate and proliferation kinetics

Once GEC conditions for in vitro culture were optimized, 1 × 10^5^ cells were seeded in triplicate in 500 μL/well of supplemented William’s E medium on plastic 12-well plates that had been previously coated with collagen type I and incubated at 37 °C, 5% CO_2_ for 7 days. A growth curve was generated to identify the exponential and stationary phases by determining the number of viable cells. Cells were collected every 24 h, centrifuged and counted using a hemocytometer with trypan blue dye at 0.4% (T8154-Sigma-Aldrich, St. Louis, USA). In addition, proliferation kinetics was measured for up to 72 h with the Cell Proliferation reagent WST-1 (5015944001-Sigma-Aldrich, St. Louis, USA). Thus, 1 × 10^4^ GECs were seeded on 96-well plates that had been previously coated and cultured as described above. Cells were harvested at 24, 48 and 72 h, for which 10 μL reagent WST-1 was added to each well and incubated at 37 °C, 5% CO_2_ for 2 h. All samples were analyzed using the iMark Microplate Reader (Bio-Rad, Hercules, CA, USA) at 540 nm. These experiments were repeated twice under the same conditions.

### Hematoxylin and eosin staining (H&E)

GEC morphology was assessed by H&E staining. 1 × 10^4^ cells were harvested from a seven-day culture monolayer, then seeded onto microscope slides and incubated at 37 °C, 5% CO_2_ for 24 h. The slides were dipped in Mayer’s hematoxylin solution filled Coplin jar for 30 s and rinsed twice with PBS 1X for 1 min each. Then, a 1% eosin Y stock solution was added for 30 s. An Eclipse 2000 microscope was used for imaging (Nikon, Tokyo, Japan).

### Mucin detection in GECs by periodic acid-Schiff (PAS) staining

GEC phenotype was confirmed by the Periodic Acid-Schiff (PAS) staining in cell cultures collected on days 0, 7 and 15. 1 × 10^4^ cells were harvested at each time point, seeded onto microscope slides that had been previously coated with collagen type I and incubated at 37 °C, 5% CO_2_ for 24 h. Then, GECs were fixed with a 4% paraformaldehyde solution in phosphate-buffered saline (PBS) for 15 min and washed in PBS. The slides were treated in a solution of 0.5% periodic acid for 5 min and stained with Schiff’s reagent for 15 min. After removing Schiff’s reagent, the slides were rinsed with running tap water for 10 min. Finally, cells were counterstained with a hematoxylin solution for 5 min. All steps were performed at room temperature (RT). A uniform reddish-purple cytoplasm was considered positive for PAS staining. Imaging was made using a Nikon Eclipse 2000 microscope.

### RT- PCR amplification of MUC1 and MUC20 genes

The expression of mucins 1 and 20 genes (*MUC1, MUC20*) in GECs was detected using RT-PCR. Total cellular RNA was isolated from 1 × 10^5^ GECs harvested on days 0, 7 and 15. Cells were first washed twice with PBS and centrifuged at 80 *g* at RT for 10 min. 1 mL RiboZol RNA extraction reagent (VWR Life Science, Radnor, PE, USA) was added to 1.5 mL tubes and RNA extraction was performed following the manufacturer’s instructions. Purified RNA was measured using NanoDrop 2000C (Thermo Fisher Scientific, Waltham, MS, USA). The isolated RNA was reverse transcribed and amplified sequentially using the OneTaq One-Step RT-PCR kit (E5315S - New England Biolabs MA, USA) with 0.4 microliters of each primer and 250 ng of total RNA in a 20 microliters reaction mixture volume, according to manufacturer’s instructions. Reverse transcription was carried out at 48 °C for 20 min, followed by an initial denaturation at 94 °C for 1 min. cDNA amplification of *MUC1* and *MUC20* genes included 40 cycles of denaturation at 94 °C for 15 s and annealing at 58 °C for 30 s, in addition to an extension at 68 °C for 30 s and a final step at 68 °C for 5 min. RT-PCR amplicons were confirmed by adding 2% agarose gel stained with SYBR Safe DNA Gel Stain (S33102-Thermo Fisher Scientific, USA). *MUC1* and *MUC20* primers as target genes and β-actin (*ACTB*) as internal control were designed for covering exon-exon junctions based on annotated sequences of swine (*Sus scrofa domesticus*). Candidate gene primers were selected by bioinformatics analysis using Primer3 software (v. 0.4.0 at http://primer3.ut.ee) and checked for specific alignments using Primer-BLAST, the NCBI online tool (https://www.ncbi.nlm.nih.gov/tools/primer-blast/) [24]. Primers and RT-PCR amplification products are listed in Table 1.

**Table 1:** List of gene primers, annealing temperature and amplicon size for *Sus scrofa domesticus* gene amplification used in RT-PCR protocols

Target Gene	RefSeq.	Sense 5′-3’	Antisense 5′-3’	Tm (°C)	Size (bp)
***MUC1***	XM_021089730.1	GACGGGCTTCTGGGACTCTTTTA	TGCTCATAGGGGTTCCGTTTGGTA	58	437
***MUC20***	NM_001113440.1	GACCTCACTGACCCCACAGT	CTGATGTACGTGGGAACCT	58	341
***ACTB***	AY550069.1	GGCACCACACCTTCTACAAC	GAGTCCATCACGATGCCAGT	58	208

### MUC1 cDNA sequencing

Amplified *MUC1* RT-PCR products were separated by electrophoresis in 2% agarose gel and purified using a commercially available QIAquick Gel Extraction Kit (Qiagen, USA), following the manufacturer’s instructions. Nucleotide sequences were directly determined from two strands by automated Sanger dideoxy sequencing using a genetic analyzer (Macrogen Inc., South Korea) and primers described in Table 1. A sequence alignment of *MUC1* was carried against *Sus scrofa domesticus* (GenBank Accession number XM_021089730.1) by the ClustalW alignment algorithm of the MegAlign software (Lasergene 15.0, DNASTAR, USA).

### Cytokeratin detection by immunofluorescence

The expression of cytokeratin-18 (CK-18) was detected by immunofluorescence to confirm the epithelial GEC phenotype. 4 × 10^4^ cells/wells were cultured on pre-coated collagen type I on sterile glass microscopic slides and incubated at 37 °C, 5% CO_2_ for 24 h. Then, cells were fixed with 4% paraformaldehyde in PBS for 15 min at RT, washed three times in PBS 1X and permeabilized in 0.02% Triton X-100 for 20 min. GECs were blocked with 5% BSA and 2% goat serum in PBS for 1 h at RT. Thereafter, GECs were incubated at RT for 1 h using the CK-18 primary antibody (sc- 32329-Santa Cruz Biotechnology Dallas, TX, USA), diluted 1:100 in PBS with 5% BSA, washed three times and then incubated at RT for another hour with the Alexa Fluor 555 goat anti-mouse secondary antibody (A-21422, Thermo Fisher Scientific, USA), diluted 1:500 in PBS with 5% BSA. After washing the slides three times in PBS 1X, the samples were treated using the UltraCruz Aqueous Mounting medium with DAPI (sc-24941, Santa Cruz Biotechnology Dallas, TX, USA). Human epithelial cell lines, known as HeLa cells (ATCC® CCL-2), and human embryonic kidney 293 T cell lines (ATCC® CRL-3216) were used as positive and negative controls, respectively. The slides were analyzed using the EVOS FL Cell Imaging Station (Thermo Fisher Scientific, USA).

### Epithelial markers detection by immunohistochemistry

The expression of additional epithelial markers such as transmembrane mucin 1 (MUC1), also known as epithelial membrane antigen (EMA), and cytokeratin cocktail (AE1/AE3) were detected by immunohistochemistry methods. Cells were fixed with 4% paraformaldehyde in PBS 1X, treated with 0.1% Triton x-100 in PBS for 15 min and blocked with 2% BSA in PBS at RT for 2 h. Then, primary anti-EMA antibodies (MS-348-P, Thermo Scientific, USA) and anti-AE1/AE3 (MA5–13156, Thermo Scientific, USA) were incubated with cells at 4 °C for 16 h at 1:100 dilution. Finally, the slides were washed in PBS and incubated with an HRP conjugated goat anti-mouse IgG secondary antibody at RT for 45 min. Immunocytochemical staining was performed using an avidin-biotin complex peroxidase standard staining kit. HeLa and AGS cell lines (ATCC CRL-1739) were used as positive controls for AE1/AE3 and MUC1 markers, respectively, whereas hematopoietic U-937 cell lines (ATCC® CRL-1593.2™) were used as a negative control. Imaging was made using a Nikon Eclipse 2000 microscope (Nikon, Tokyo, Japan). All experiments were performed in triplicate.

### Contamination with mycoplasma spp.

To confirm the absence of *mycoplasma spp* contamination, 1 × 10^4^ GECs were fixed with a 4% paraformaldehyde solution in PBS for 15 min, washed three times in PBS and treated using a consistent size at RT for 10 min. Stained cells were analyzed using a fluorescence microscope to detect the presence of small nuclear bodies in the cell cytoplasm associated with Mycoplasma infections.

### Statistical analysis

IBM SPSS Statistics v21 was used for data collection and statistical analysis. All data were expressed as mean values. Error bars represent ± standard deviation (SD).

## Results

### Cell isolation and establishment of GEC cultures

GEC isolation was possible through an optimized mechanical and enzymatic method maintaining high cell viability (> 95%) after suspending tissues from the fundic gland region of the pig stomach in a digestion medium. Neither the presence of mucus on cell cultures nor the disparity in pH of culture medium was observed. Four different proliferation media were assessed to determine the best culture conditions for GECs. To this end, cells cultured in DMEM-HG, DMEM/F12, RPMI 1640 and William’s E media, supplemented with a combination of growth factors and FBSi, were evaluated in terms of cell viability and adhesion (a minimum of 20–30% of cell confluency during the first 48 h of incubation) and cell proliferation (about 80–100% of cell confluency on day 10 after seeding). The results suggested that cells cultured in DMEM-HG, DMEM/F12 and RPMI 1640 media could not achieve the required GEC adhesion and proliferation. Moreover, the microscopic inspection of GEC cells revealed a necrosis-like form of programmed cell death such as nuclear condensation, loss of plasma membrane integrity and ghost cells, as well as abundant cellular debris (unpublished data). In contrast, William’s E medium facilitated the establishment of GEC cultures, which are characterized by initial cell adhesion, cell clustering and proliferation and the absence of necrotic-like bodies (Table 2).

**Table 2:** Summary of GEC characteristics cultured in different cell media

Cell culture medium	Adhesion cells	Proliferation rate	Necrotic features
DMEM-HG	–	–	+
DMEM/F12	–	–	+
RPMI 1640	–	–	+
William’s E	+	+	–

+ Presence, − Absence

**Fig. 1:**
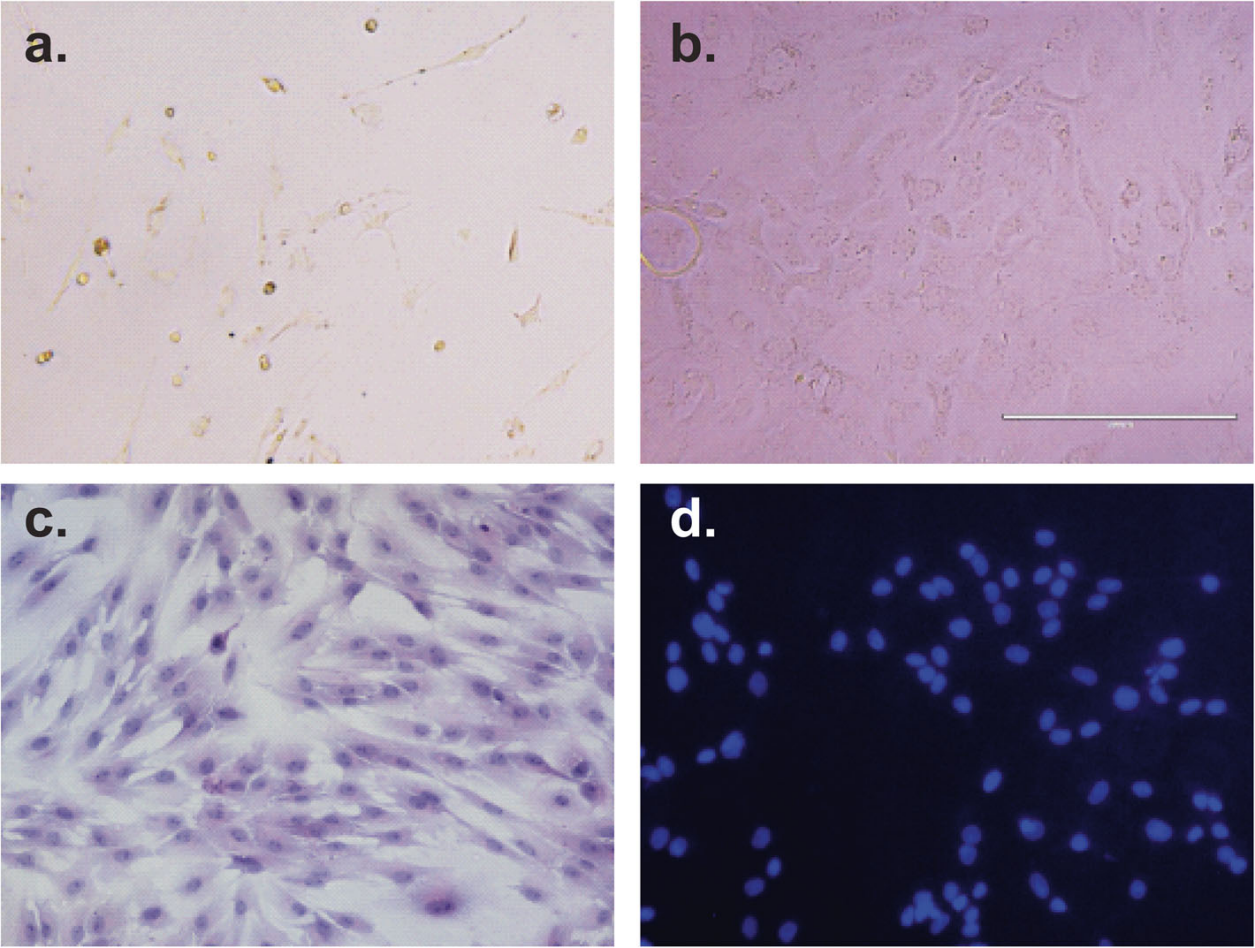
Microscopic features of GECs cultured in a William’s E medium. **a.** GECs on day 3 after seeding reaching initially 20% confluency. **b.** GECs after 10 days of culture reaching 100% confluency. **c.** GECs stained with hematoxylin and eosin stain. **d.** GECs stained with DAPI for the exclusion of *Mycoplasma spp.* contamination. Optical microscope images were taken at 20x magnification. The figure shows a representative experiment

Furthermore, GECs reached about 20 and 100% of cell confluency in William’s E medium on day 3 and 10 after seeding (Fig. 1a and b). GECs with H&E staining maintained the morphology of epithelial cells such as a prominent nucleolus, polygonal shape and consistent cell size (Fig. 1c). In addition, GECs were tested for *Mycoplasma spp* contamination by DAPI fluorescent dye. The microscopic evaluation revealed that staining GEC cytoplasm had no nuclear bodies, confirming the absence of infections (Fig. 1d). Finally, GECs were cultured for up to 2 months, preserving their epithelial morphology and growth rate (unpublished data). In conclusion, these results suggested that William’s E medium provided the best conditions for establishing and culturing primary GECs from the fundic gland region of the pig stomach.

### GEC viability and growth rate

**Fig. 2:**
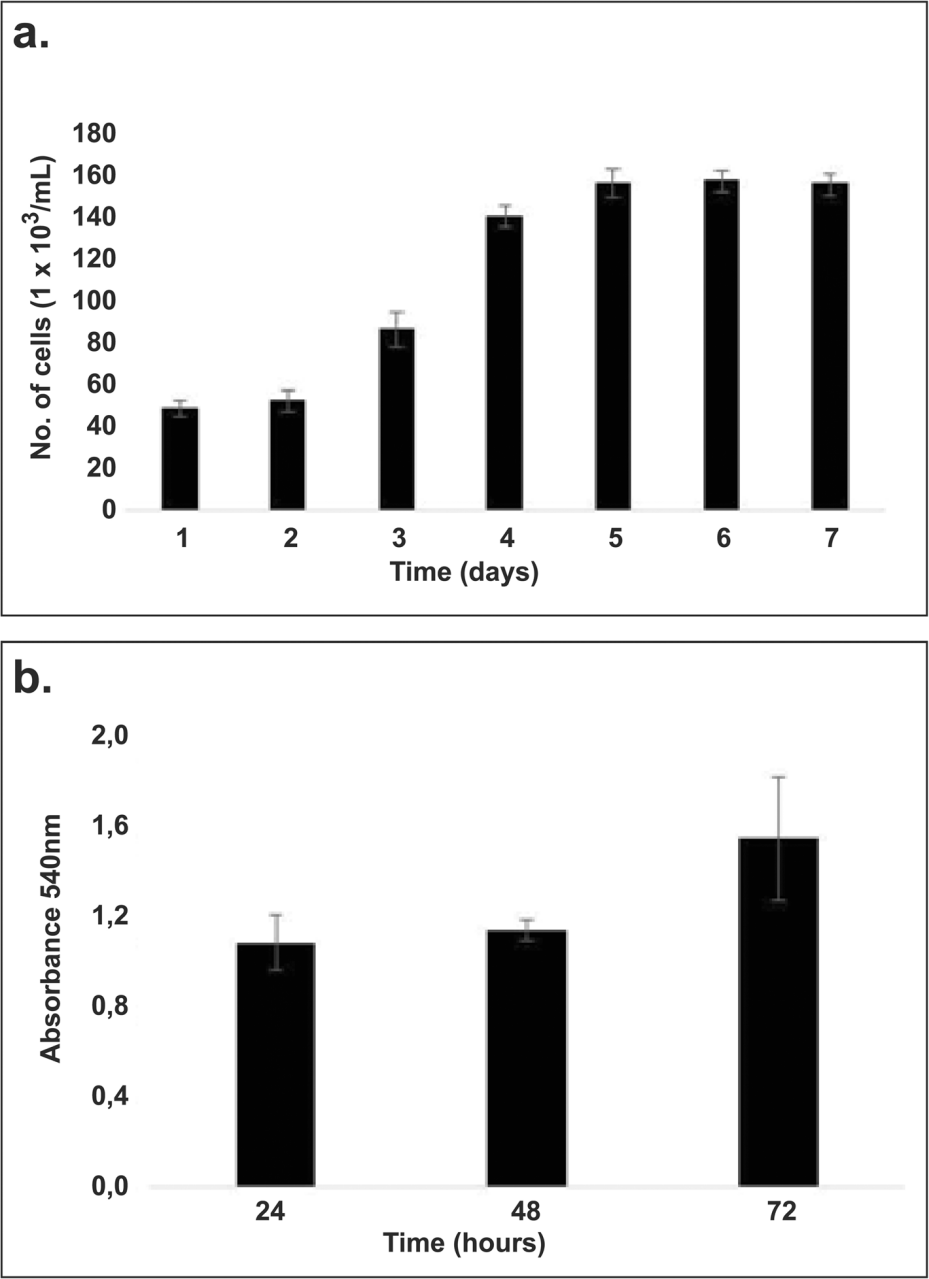
Growth and cell viability of GECs cultured in a William’s E medium. **a.** GEC cultures were assessed for seven consecutive days using trypan blue, then cell growth and proliferation kinetics curves were drawn. **b.** GECs cultured in 96-well plates were exposed to WST-1 reagent for viability determination by optical density using ELISA plate readers. The figure shows the mean ± SD of the number of cells at OD 540 nm

The WST-1 assay assessed cell viability, having the same levels during the first 48 h after culturing. However, an increase in optical density due to GEC proliferation was observed at 72 h. Such increase is correlated with the findings of trypan blue dye experiments (Fig. 2b), in which a twofold increase in the number of cells was observed on day 4 (exponential phase). Whether cell count remained unchanged (stationary phase) on days 6 and 7 with a 90–100% confluent monolayer, contact-dependent growth inhibition in primary cultures was observed (Fig. 2a). Based on these cases and the data obtained, it was possible to confirm that primary GEC isolates from pig stomach preserved the standard characteristics of commercially available immortal cell lines such as stable cell viability, exponential and stationary growth phases, and contact-dependent growth inhibition.

### Mucin expression in GECs

**Fig. 3:**
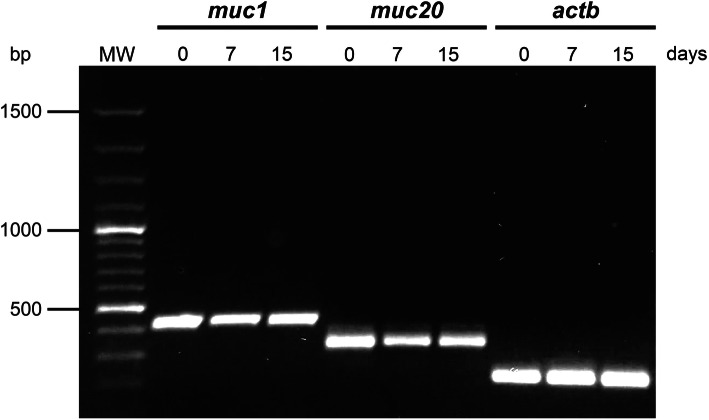
Expression of *MUC1* and *MUC20* genes in GECs. 1 × 10^5^ GECs were used for RNA extraction on days 0, 7 and 15, and then reverse transcribed to cDNA for amplification of *MUC1*, *MUC20* and *ACTB* genes. RT-PCR products were separated in 2% agarose gel stained with SYBR safe. The figure shows a representative experiment

**Fig. 4:**
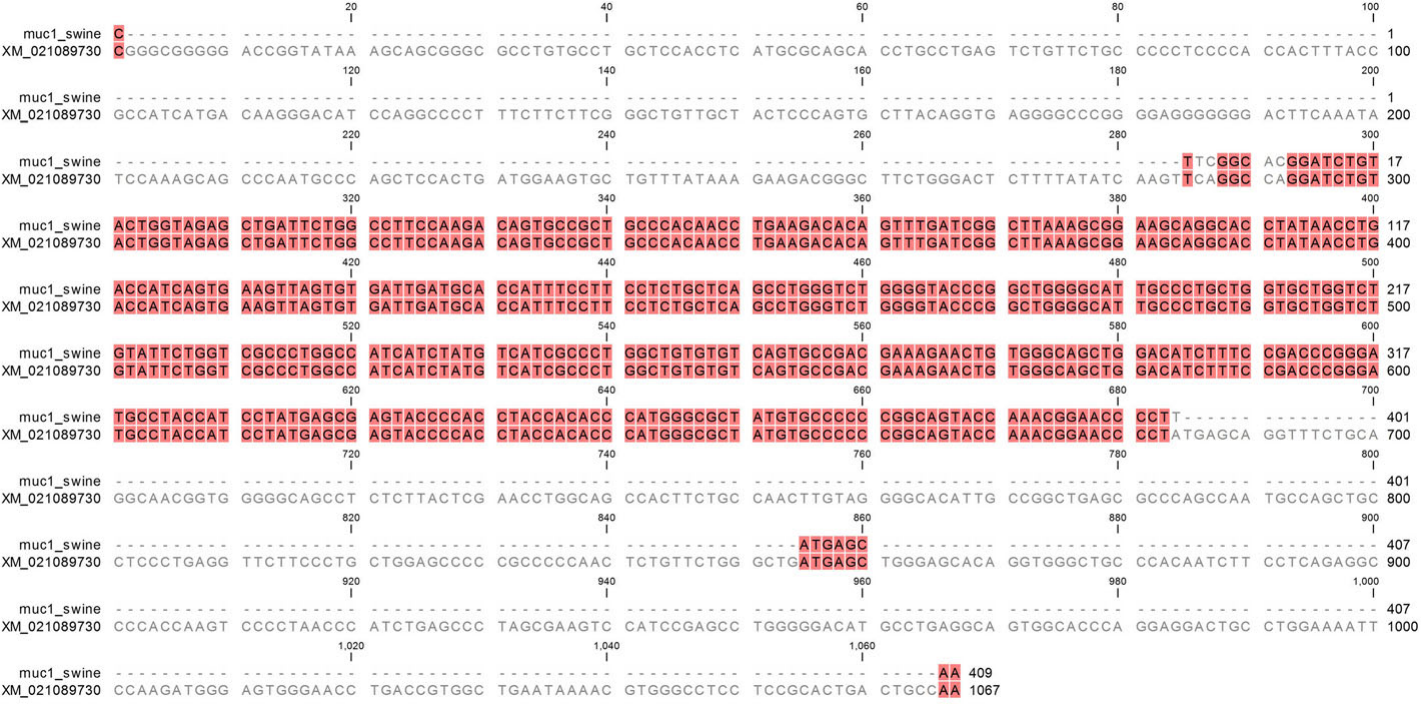
Sequence analysis of *MUC1* gene from GECs (GenBank accession number MW321489). RT-PCR amplicon (437 bp) was sequenced and aligned with the swine *MUC1* reference sequence (GenBank accession number XM_021089730.1) by ClustalW. Background colored nucleic bases indicate sequence homology

Mucins are glycoproteins covering the gastric epithelium, which are highly expressed in the stomach [25]. To confirm the phenotype of GECs isolated from the fundic glands of pig stomach, *MUC1* and *MUC20* genes were detected by RT-PCR on days 0, 7 and 15 after culturing. Genes encoding β-actin (*ACTB*) were also amplified as an internal control. Cellular RNA allowed RT-PCR amplification of *MUC1* and *MUC20* genes multiples times*.* Similar band intensity was observed in agarose gel, confirming constant expression of mucins regardless of the length of time in culture. The amplification of internal positive controls (using a housekeeping gene) was also detected, suggesting that the presence of target genes was stable within the samples (Fig. 3). In addition, the sequence analysis of *MUC1* gene from our study (GenBank accession number MW321489) shared 100% homology with *Sus scrofa domesticus* (GenBank accession number XM_021089730.1), confirming the specificity of the molecular biology assay (Fig. 4).

**Fig. 5:**
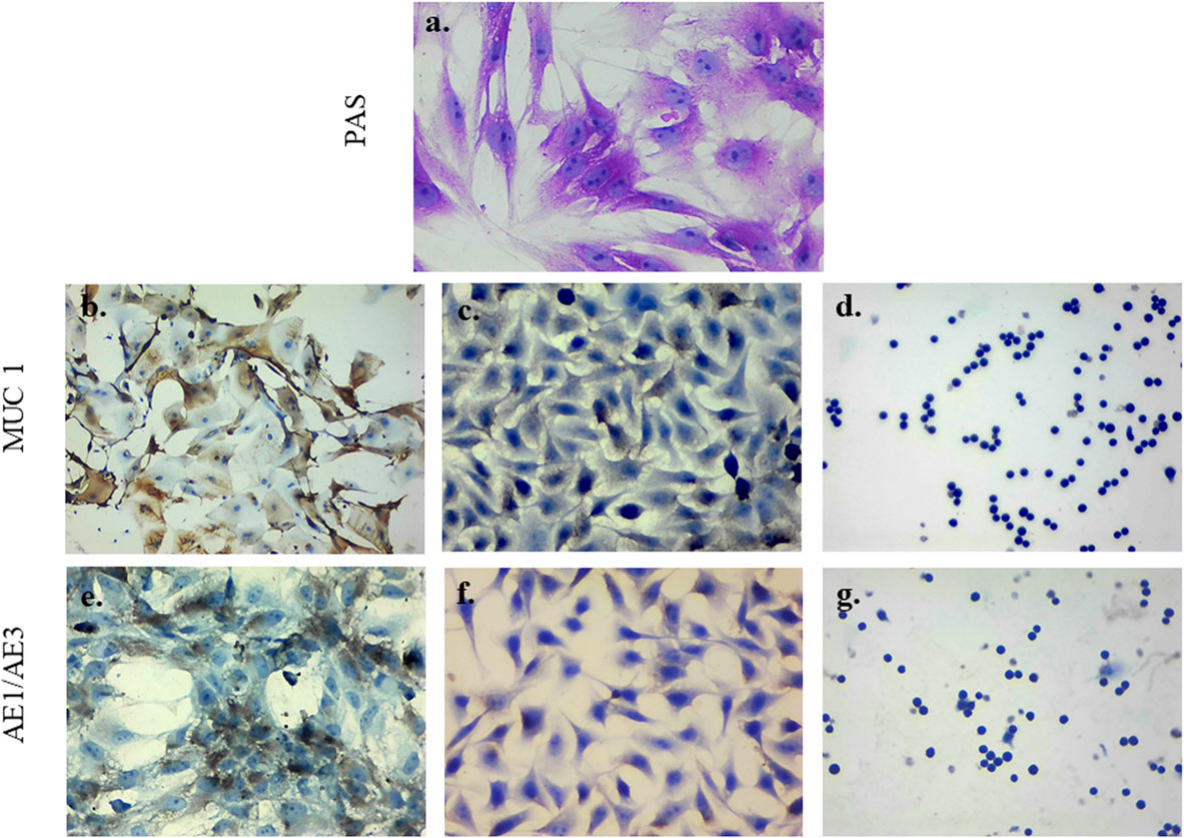
Mucin production and epithelial phenotype in GECs. **a.** GEC were stained with PAS (reddish-purple staining indicates the presence of neutral mucins), **b.** MUC1 on GECs shows homogeneous cytoplasmic staining mainly located in the cell membrane, **c.** AGS cell lines (mucin positive control) and **d.** U-937 cell lines (negative control) e**.** Pan-cytokeratin markers AE1/AE3 on GECs: a positive fibrillar staining pattern distributed in the cytoplasm is observed, **f.** HeLa cell lines (positive control) and, **g.** U-937 cell lines (negative control). Images were visualized using conventional light microscopy. Images were taken at 20x magnification

In addition, to determine the expression pattern of neutral mucins, PAS-stained GECs were examined under an optical microscope. The evaluation of PAS-stained GECs showed a reddish-purple color in the cytoplasm, which is a common characteristic of mucus-secreting cells. Cells also showed a regular epithelial-like shape and nucleus shape and positioning (Fig. 5a). These data led to the conclusion that cell culture from pig stomach tissue is very similar to GECs as mucin genes and protein expression were constantly detected. Moreover, William’s E medium supplemented with specific growth factors supported in vitro expression and synthesis of these epithelial glycoproteins.

### Expression of epithelial cell markers in GECs

**Fig. 6:**
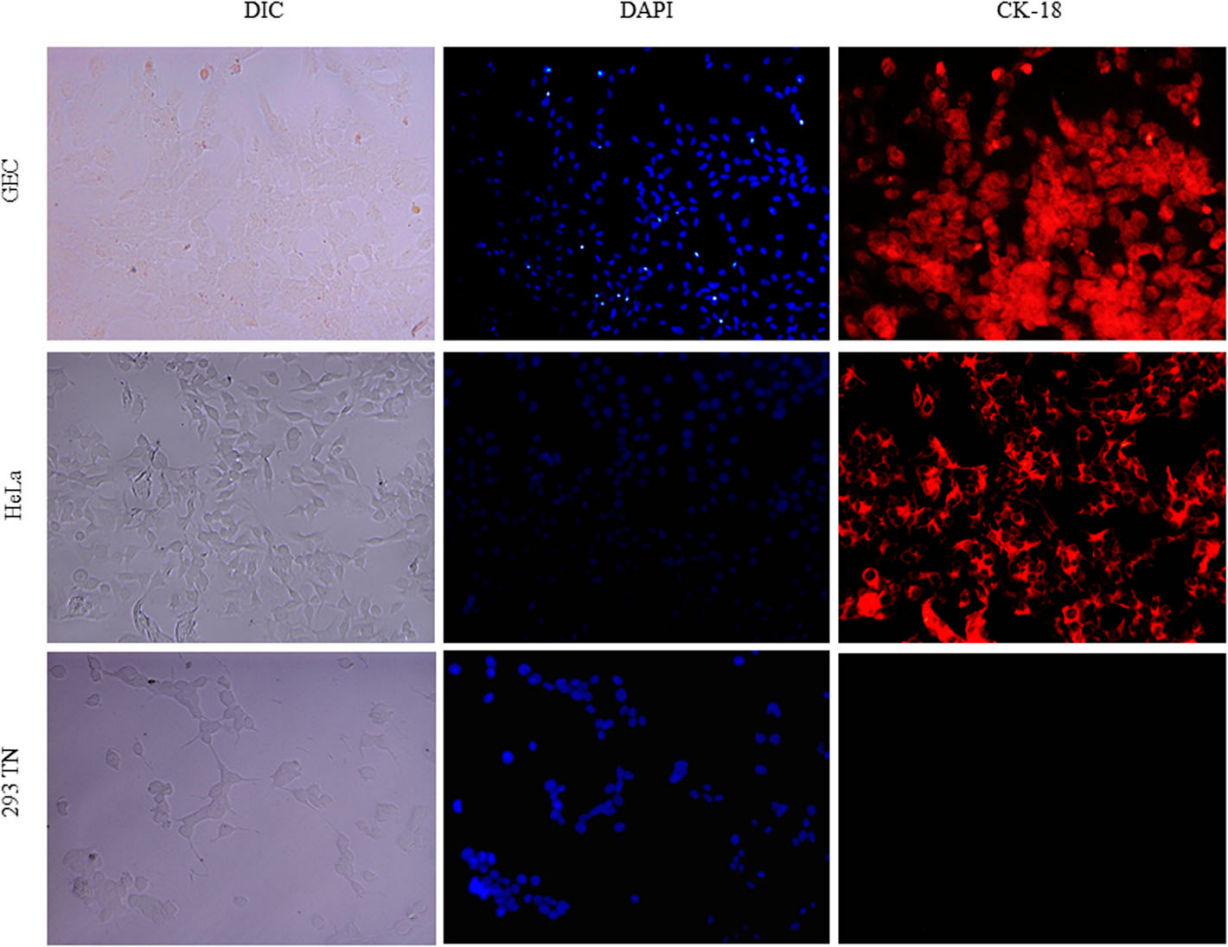
Immunofluorescence staining for epithelial-specific marker CK-18 in GECs. DAPI staining (blue, middle panel) identified cell nuclei for each cell line. HeLa and 293 T cell lines were used as positive and negative controls for the CK-18 epithelial marker, respectively. (DIC: differential interference contrast). Magnification: 20X

The expression of three different epithelial cell markers, namely MUC1, AE1/AE3 and CK-18, were evaluated by immunohistochemical and immunofluorescence methods, respectively, to verify the nature of GECs. MUC1 is a cell surface-mucin expressed on the apical membrane surface of most mucosal epithelial cells, including the gastric mucosa. Cytokeratin is found in the form of filaments in the cytoplasm, which is usually associated with the cytoskeleton of epithelial cells. AE1/AE3 detect several cytokeratins at the same time except for CK-18 [26]. The optic evaluation of GECs exhibited brown homogeneous staining mainly over the cell membrane, which corresponds to MUC1 expression. About 80% of GECs were reported to be positive for this epithelial marker (Fig. 5b). In addition, the expression of cytokeratin AE1/AE3 was confirmed by the presence of grey-colored stains distributed as filaments in the cytoplasm with 80 to 90% of positive target cells (Fig. 5e). Fluorescence-positive cells for CK-18 were also observed in 80–90% of the examined microscopic fields with a cytoplasmic pattern. Similarly, DAPI nuclear counterstain demonstrated the integrity and epithelial-like shape of GECs (Fig. 6). These results confirmed that GECs isolated from the fundic glands of the pig stomach maintained epithelial biomarkers and mucin expression genotype.

## Discussion

Establishing epithelial cell cultures derived from the fundic gland zone of pig stomach tissue has been proposed as a tool for studying different types of gastric disease in both humans and swine. In this regard, several works have examined the usefulness and applicability of swine models, in comparison to murine models, as swine tissue architecture and pathophysiology features are similar to those of humans. For example, several research studies on cardiovascular diseases, organ transplantation, diabetes, wound healing and cancer, among others, have been performed on wild pig species, which have provided conclusive data for translational research [8]. Furthermore, porcine airway and intestinal epithelial cells have been used for studying major emerging zoonotic diseases such as influenza viruses [27]. Gastric parietal cells have been also used for determining cell viability and function in *H. suis* [19]. Similarly, the establishment of GEC cultures seeks to shed light on the biological process associated with the development of many diseases such as gastric disorders in humans and swine.

Several protocols have been developed for the isolation and culture of primary normal cells from human and porcine gastric mucosa. For example, non-enzymatic strategies have been developed in which fragments of gastric tissue are allowed for dry incubation so that epithelial cells can adhere to the surface that had been previously treated with a fibronectin solution [28]. Similarly, the isolation of gastric cells from human tissues have been performed using a MatriSperse Cell Recovery Solution, a non-enzymatic treatment that depolymerizes the extracellular matrix of the basement membrane to isolate pure epithelial cells [29]; other authors have used a combination of enzymatic and mechanical treatments to remove the connective tissue and the underlying muscle layers from the gastric mucosa, followed by tissue digestion using different enzyme mixtures [30–32]. The digestive enzymes include collagenase type IV/Deoxyribonuclease (DNase) [30], collagenase type II/dispase [31] and collagenase type IA/dispase [32]. Finally, enzymatic protocols for the isolation of GECs in pigs also benefit from initial mechanical removal of thick mucus layers and the isolation of the gastric mucosa from the underlying submucosa and tunica muscularis [19–22]. Digestion of porcine gastric tissue has been reported to be made using enzymes such as collagenase A [21, 22] and collagenase type 1 [19] or in combination with pronase [20] and dispase [19].

A new methodology is described here for establishing primary cell cultures from fundic glands of the pig stomach. This protocol is an adaptation of culture methods used to isolate epithelial cells and generate primary cultures from the human and pig stomach by including the use of protease inhibitors to neutralize the effects of proteolytic enzymes and antioxidants so that cellular redox environment can be maintained. The proposed method is based on two different approaches: mechanical and enzymatic dissociation. Collagenase type I, dispase II, protease inhibitors and antioxidants together showed improved performance in terms of GEC viability and complete tissue dissociation during the incubation period. Collagenase type I is widely used in digest protocols and cell culture due to its protease potential for disassembling collagen fibrils from the connective tissue. Furthermore, collagenase type I led to the isolation of fibroblasts [33] and the culture of small intestinal epithelial cells [34]. Dispase II is a collagenase type IV with a mild proteolytic activity that has also been used in animal tissues for isolating labile primary cells such as porcine urothelial cells, lymphatic and embryonic endothelial cells, stem cells and Schwann cells [35–38]. In addition, protease inhibitors (STI and BSA) are recommended to protect cells from a non-specific proteolytic and antioxidant (DTT) activity in a cellular redox environment [39, 40]. Therefore, the compounds added to the digestion mixture maintained higher GEC viability and adhesion.

In addition, the highest performance in cell culture was obtained through William’s E medium supplemented with mitogens in comparison to RPMI 1640, DMEM-HG and DMEM/F12. The above was due to the induction and maintenance of cell adhesion and proliferation, which allowed cell growth for up to 2 months. William’s E medium was originally developed for isolating and growing hepatocyte cells [41] and, to the best of our knowledge, this medium had not been used for isolating GECs before. This medium contains higher glucose concentrations and essential amino acids and vitamins, among others, that lead to the inhibition of apoptosis and the support of long-term cell cultures. In addition, GECs had been previously isolated using well-known media such as DMEM/F12 [19], MEM [22] and RPMI 1640 [21].

Furthermore, cell culture media should indeed have mitogens to support the growth and proliferation rate of primary culture cells and maintain tissue-specific features [42]. In this study, William’s E medium was supplemented with mitogens such as EGF and insulin. The EGF has been observed to be involved in a variety of physiological responses such as cell survival as well as cell proliferation and differentiation in several tissues. It has been also described to have an anti-apoptotic effect in gastric epithelial cells infected by *H. pylori* [43], in addition to the control of mucus production and airway epithelium repair after injury [44]. Additionally, insulin helps metabolize glucose and amino acids into cells to stimulate the growth and proliferation of rabbit gastric epithelial cells [45]. There is evidence that the combination of insulin and EGF improves the proliferation of human gastric epithelial cells [46]. Therefore, isolating GEC cells in a William’s E medium supplemented with mitogens (EGF and insulin) is then proposed, which improves cell viability and proliferation as well as phenotype preservation, thus making this model more reliable for long-term experiments.

Cell-matrix adhesion to the extracellular matrix during the establishment and maintenance of primary culture cells is a major step to control cell viability and proliferation [42]. Several extracellular matrix coatings have been described to promote cell adhesion in GECs, including a glass plate coated with fibronectin or collagen type IV from human placenta [20] and Matrigel [19]. In this study, GECs were seeded in bovine collagen type I to maintain cell proliferation rates in culture for up to 2 months, preserving their epithelial morphology and cellular architecture.

It was possible to observe that isolated GECs genotypically expressed gastric epithelial markers. In this regard, the expression of *MUC1* and *MUC20* genes was confirmed using RT-PCR. Mucins are glycoproteins secreted by specialized epithelial cells that are located in the lumen of organs of the digestive, respiratory and reproductive tracts. These cells provide lubrication and maintain epithelial integrity [47]. MUC1 and MUC20 are membrane-bound mucins constitutively present in epithelial cells that have been used as makers in GECs. Alterations in mucin expression are related to *H. pylori* infections and gastric cancer [25, 48, 49]. Thus, the expression of *MUC1* and *MUC20* genes in GECs proves that the gastric epithelial phenotype of these cells was conserved.

The expression of epithelial markers was also identified to confirm the epithelial phenotype of GECs by immunohistochemical detection of MUC1 and AE1/AE3, and immunofluorescence detection of CK-18. Positive staining was found in GECs for all markers. MUC1 was restricted to the cell membrane surface of GECs, whereas pan-cytokeratin markers and CK-18 were scattered throughout GECs. Although these markers are normally expressed in GECs, a higher marker expression can be a sign of human cancer [50]. Through all these data, the functional phenotype of GECs could be confirmed, which is why it is necessary to establish a new method for isolating swine GECs.

Finally, it is widely known that the use of human tissues, especially those from solid organs, has significant clinical and ethical implications that lead to the lack of biopsies available for basic research. Therefore, the use of porcine tissue samples, as these share some common properties of human organs, offers numerous advantages given the recent growing interest in non-rodent animal models for the study of human disease. This protocol contributed to the development of translational research in acute and chronic gastrointestinal diseases in humans and swine.

## Conclusions

A new combined mechanical and enzymatic procedure is reported herein for isolating and culturing GECs based on the evaluation of different components of cell culture media. William’s E medium supplemented with mitogens (FBSi, EGF and insulin) provides the best conditions for GEC proliferation and preservation of functional tissue-specific features such as mucin production and expression of epithelial cell markers (MUC1, CK-AE1/AE3 and CK-18) in the long term. The research methodology here discussed has important biological applications to study physiological and pathological characteristics in toxicological, pharmacological and microbial interactions, in addition to inflammatory gastric diseases in humans and swine.

## Data Availability

Raw data were generated at the Biomedical Research Laboratory of the University of Santander - UDES, where all tissue samples have been properly stored. Derived data supporting the findings of this study are available from the corresponding author (HBA) upon request. The datasets generated and analyzed for *MUC1* gene, during the current study, are available in the GenBank repository accession number MW321489.

## Abbreviations

*ACTB*: Beta-actin
BSA: Bovine serum albumin
CKs: Cytokeratin cocktail (AE1/AE3)
DAPI: 4′,6-diamidino-2-phenylindole
EMA: Epithelial membrane antigen
EGF: Epidermal growth factor
FBS: Fetal Bovine serum
GEC: Gastric epithelial cells
*MUC1*: Mucin 1
*MUC20*: Mucin 20
PAS: Periodic acid-Schiff stain
RT-PCR: Reverse transcription polymerase chain reaction
RT: Room temperature
